# Response

**DOI:** 10.1007/s12471-017-0958-3

**Published:** 2017-02-07

**Authors:** R. T. van Domburg

**Affiliations:** Thoraxcenter, Erasmus Medical Center Rotterdam, Rotterdam, The Netherlands

We would like to thank Drs Pluijmers and Denollet for acknowledging the importance of our findings about the complex relationship between depression and Type D personality. We also support their theory that this association could be explained by dysregulations of the neuroendocrine, autonomic nervous, and immune systems as was found in several studies.

On behalf of all authors,

R.T. van Domburg

